# Assessing the impact of short-term ozone exposure on excess deaths from cardiovascular disease: a multi-pollutant model in Nanjing, China’s Yangtze River Delta

**DOI:** 10.3389/fpubh.2024.1353384

**Published:** 2024-06-13

**Authors:** Fengxia Sun, Xing Gong, Li Wei, Yi Zhang, Ming Ge, Liling Xiong

**Affiliations:** Department of Environment Health, Nanjing Municipal Center for Disease Control and Prevention, Nanjing, China

**Keywords:** ozone, short-term exposure, cardiovascular disease, excess deaths, multipollutant model

## Abstract

**Background:**

Ozone pollution is associated with cardiovascular disease mortality, and there is a high correlation between different pollutants. This study aimed to assess the association between ozone and cardiovascular disease deaths and the resulting disease burden in Nanjing, China.

**Methods:**

A total of 151,609 deaths from cardiovascular disease were included in Nanjing, China from 2013 to 2021. Daily data on meteorological and air pollution were collected to apply a generalized additional model with multiple pollutants to perform exposure-response analyses, stratification analysis, and evaluation of excess deaths using various standards.

**Results:**

In the multi-pollutant model, an increase of 10 μg/m^3^ in O_3_ was significantly associated with a 0.81% (95%CI: 0.49, 1.12%) increase in cardiovascular disease deaths in lag05. The correlation weakened in both the single-pollutant model and two-pollutant models, but remained more pronounced in females, the older group, and during warm seasons. From 2013 to 2021, the number of excess deaths attributed to ozone exposure in cardiovascular disease continued to rise with an increase in ozone concentration in Nanjing. If the ozone concentration were to be reduced to the WHO standard and the minimum level, the number of deaths would decrease by 1,736 and 10,882, respectively.

**Conclusion:**

The risk of death and excess deaths from cardiovascular disease due to ozone exposure increases with higher ozone concentration. Reducing ozone concentration to meet WHO standards or lower can provide greater cardiovascular disease health benefits.

## 1 Introduction

Ambient air pollution has become a significant global public health concern. Ozone, one of the primary ambient pollutants, is involved in various atmospheric photochemical reactions with high chemical activity. This leads to tropospheric pollution, including the formation of photochemical smog, reduced atmospheric visibility, and direct or indirect harm to human health. Ozone is primarily produced through the photochemical reaction of nitrogen oxides (NOx) and volatile organic compounds (VOCs) under ultraviolet radiation. The diverse range of influencing factors and the uncertainty surrounding precursor sources present a challenge in efforts to control ozone pollution.

In recent decades, the global economy has experienced a significant growth spurt, resulting in a continuous increase in global tropospheric ozone concentrations. Low-latitude areas have witnessed a higher rate of ozone concentration increase compared to middle and high latitudes, such as North America and Europe (1). In China specifically, the annual average concentration of ozone has roughly shown an increasing trend during 2013–2021 (2), and since 2018 it has been consistently exceeding 100 μg/m^3^,^1^ which is significantly higher than the recommended AQG levels. In 2013, the Chinese government implemented the “Regulations on the Prevention and Control of Air Pollution” to address severe air pollution issues. Despite these efforts, persistent VOCs emissions and meteorological factors have contributed to a severe situation of ambient ozone pollution, posing significant health risks to the population (3, 4). A growing trend of studies has then linked ozone exposure to mortality in populations, particularly from cardiovascular disease (CVD) (5, 6).

CVD is the leading cause of morbidity and mortality globally. From 1990 to 2019, the number of CVD deaths steadily increased from 12.1 million to 18.6 million (7), while the number of deaths increased from 2.4 million to 4.6 million in China (8). And there is growing evidence that exposure to ozone pollution can increase CVD mortality. Quantification of the CVD mortality burden indicates that the ozone-attributable CVD deaths in 2019 were 1,467.8 thousand, and by 2050, the estimated ozone-attributable CVD deaths in Chinese adults are expected to be 1,082.4 thousand and 359.2 thousand, respectively, according to SSP 585 and SSP 126 (9). A cohort study found that a 10 μg/m^3^ increase in long-term annual average ozone exposure was associated with a 1.22 (95% confidence interval [CI]: 1.13–1.33) increase in adjusted hazard ratio for CVD deaths (10), while for short term ozone exposure, with lag 0–1 day, the odds ratio (OR) for CVD mortality ranged from 1.009 to 1.012 for each 10 μg/m^3^ increase (11). During 2015–2021, the ozone concentration in Jiangsu Province showed an increasing trend (11), and Nanjing, a key development city and the capital city of Jiangsu Province, has maintained the same increasing trend of ozone pollution as Jiangsu Province, whose cardiovascular disease burden caused by ozone has aroused widespread public concern. Additionally, in recent years, there is growing evidence that other air pollutants are also markedly associated with CVD deaths (12–14). The single-pollutant approach may misestimate the strength and direction of this association. Therefore, it is more reasonable to employ a multi-pollutant model to comprehensively assess the effects of exposure to ozone on CVD deaths.

The present study collected data on major air pollutants and population health surveillance in Nanjing during the period of 2013–2021, with the aim of assessing the excess risk of CVD deaths due to ozone pollution in Nanjing by using a multi-pollutant model, and estimating the number of excess deaths by using exposure-response coefficients. The study can effectively and accurately quantify the extent of CVD deaths due to ozone exposure and provide a basis for planners and policy makers to make relevant decisions.

## 2 Materials and methods

### 2.1 Study population and outcome

We collected daily death data covering the period of January 1st, 2013 to December 31st, 2021 in Nanjing from the “China Disease Surveillance System Death Surveillance Network Reporting Database”. The causes of death were classified based on the tenth revision of the International Statistical Classification of Disease (ICD-10), with cardiovascular disease being coded as I00-I99.

### 2.2 Meteorological and pollution data

Daily meteorological data including daily mean temperature, and mean relative humidity were collected from the China meteorological sharing service system network^2^ during the corresponding mortality data period. Daily air pollution data including 24-h average concentrations of fine particular matter (PM_2.5_), inhalable particles (PM_10_), nitrogen dioxide (NO_2_), sulfur dioxide (SO_2_), and carbon monoxide (CO), and daily maximum 8-h mean concentrations of ozone (O_3–8h_) were obtained from the Nanjing environmental monitoring center.

### 2.3 Statistical analysis

#### 2.3.1 Correlation analysis

To explore the correlation between the daily mean values of each factor, including meteorological factors (temperature and relative humidity) and six air pollutants (PM_2.5_, PM_10_, SO_2_, NO_2_, CO, and O_3_), and considering that these data may not conform to a normal distribution, a Spearman’s correlation test was performed using the “psych” R package. Moreover, the correlation coefficients were plotted using the “corrplot” R package.

#### 2.3.2 Time-series analysis

We developed a foundational model using the Generalized Additive Model (GAM) to investigate the impact of O_3_ on the number of cardiovascular disease deaths.


Yt~quasiPoisson(Ut)

Where Yt represents the count of cardiovascular diseases death at day t following a Poisson distribution. In this study, the associations of daily O_3_ concentration with cardiovascular diseases deaths were estimated utilizing GAM with quasi-Poisson regression as follows:


$$\begin{array}{l} \log\;\left(\mathrm{Yt}\right)=\beta *\mathrm{Xt}+s\;\left(\mathrm{time,df}\right)+s\;\left({\mathrm{PM}}_{2.5}\mathrm{,df}\right)\\ +s\;\left({\mathrm{PM}}_{10}\mathrm{,df}\right)+s\;\left({\mathrm{SO}}_{2}\mathrm{,df}\right)+s\;\left({\mathrm{NO}}_{2}\mathrm{,df}\right)\\ +s\;\left(\mathrm{CO,df}\right)+s\;\left(\mathrm{temperature,df}\right)+s\;\left(\mathrm{humidity,df}\right)\\ +Holiday+DOW+\alpha \end{array}$$

Where *β* indicates the exposure response coefficient, X indicates the daily O_3_ concentration. *s* denotes splines function. *Holiday* and *DOW* are two categorical variables that represent the presence of a public holiday and the day of the week, respectively. *α* represents the intercept.

In our research, we included five air pollutants in our multi-pollutant model, including PM_2.5_, PM_10_, SO_2_, NO_2_, and CO. 7 degrees of freedom (*df*) per year was used to control for the long-term trend and seasonality of daily deaths, while the *df* of temperature, and relative humidity were 3 factors in our research. The lag effects associations between the O_3_ and cardiovascular deaths were assessed, with a maximum lag period of 7 days. Lag1 presents single day lag and lag01 presents moving average over 2 days.

Excessive risk (ER) presents a percentage increase or decrease in daily deaths for every 10 μg/m^3^ increase in ambient O_3_ concentration.


ER%=[exp(β∗10)−1]∗100%

Based on the model parameters obtained from the main analyses, we conducted additional subgroup analyses to explore potential modifications of the effects by age (≤65 years, >65 years), sex (male, female), seasonal (warm period: from April to September; cold period: from October to March) factors. To test for statistically significant differences between effect estimates of the potential effect modifier strata, we calculated the 95% confidence interval as presented below:


$$\left(Q_{1}-Q_{2}\right)\pm 1.96\sqrt{SE_{1}^{2}+SE_{2}^{2}}$$

Where Q_1_ and Q_2_ represent the estimates for the two categories, while SE_1_ and SE_2_ represent their respective standard errors.

#### 2.3.3 Excess death calculation

The exposure-response relationship coefficient between ozone and cardiovascular diseases obtained by the time-series analyses in Nanjing, China was used to estimate the endemic excess death attributed to ozone exposure. We calculate the excess death based on the following:


$$\Delta X=X*\left\{1-\frac{1}{\exp\left[\beta \times \left(C-C_{0}\right)\right]}\right\}$$

Where Δ*X* is the excess death; *X* is the daily death of cardiovascular diseases in Nanjing, China; β is the exposure-response relationship coefficient. In this study, we selected the largest effect with lag days to estimate excess death; C is the daily maximum 8-h mean ozone concentration; $C_{0}$ is reference concentration. The WHO air quality guidelines (AQG) were developed to provide recommendations for mitigating the health impacts of air pollution. These guidelines have set 100 μg/m^3^ as the annual standard of O_3–8h_ concentration globally. To evaluate the potential reduction in excess death, we considered the year 2013 as the baseline scenario and assessed the avoidance of excess death in line with the attainment of the WHO guidelines, referred to as the AQG scenario.

In China, the National Environmental Quality Standards (CNAAQS Grade II) have set the limit for O_3–8h_ concentration at 160 μg/m^3^. We developed and compared scenarios based on these standards to calculate the excess death and the potential avoidable excess death resulting from the reduction in ambient ozone concentration.

### 2.4 Sensitivity analysis

We conducted sensitivity analyses to ensure the robustness of our results by examining the impact of variations in the models, specifically in terms of the degree of freedom and lag days. The association between O_3_ and CVD deaths was considered robust if the effect estimates yielded by these models did not exhibit significant differences.

All analyses were performed with R software (version 4.0.2), using the *mgcv* and *ggplot2* packages. Statistical significance was defined as two-sided *p* < 0.05.

## 3 Results

### 3.1 Descriptive statistics

#### 3.1.1 Ambient pollutants and meteorological factors

**Figure 1:**
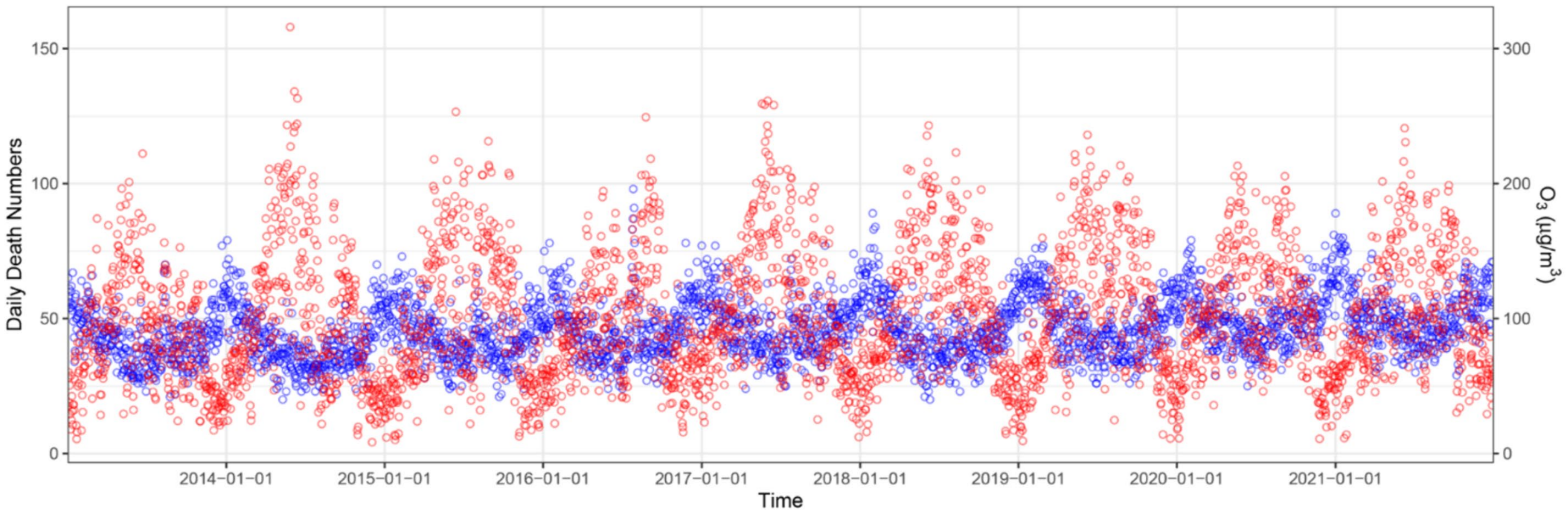
Time series of ozone concentration and daily deaths of cardiovascular disease in Nanjing, China, from January 1st, 2013 to December 31st, 2021. Red cycle, the concentration of ozone; blue cycle, daily death number of cardiovascular disease.

As shown in Figure 1, the daily average concentration of O_3–8h_ ranged from 8.40 μg/m^3^ to 316.00 μg/m^3^, with a mean concentration of 99.93 μg/m^3^. The median ambient O_3_ concentration (O_3–8h_) in Nanjing from 2013 to 2021 was 80.40 μg/m^3^, 87.80 μg/m^3^, 88.20 μg/m^3^, 84.85 μg/m^3^, 95.00 μg/m^3^, 98.29 μg/m^3^, 101.79 μg/m^3^, 100.43 μg/m^3^, and 94.71 μg/m^3^, respectively. Figure 1 depicts the temporal distribution of ozone concentration, which peaked during summer. The annual average concentration of PM_10_, PM_2.5_, SO_2_, NO_2_, CO were 86.79 μg/m^3^, 48.93 μg/m^3^, 16.44 μg/m^3^, 42.86 μg/m^3^, 0.91 mg/m^3^. The mean daily temperature and relative humidity were 16.94°C and 72.89%. From 2013 to 2021, the number of days with ozone concentration exceeding the CAAQS Grade II (China Ambient Air Quality Standards) threshold of 160 μg/m^3^ was 425, with an excessive rate of 12.93%. Further details about other air pollutants and meteorological data can be found in Table 1.

**Table 1:** Descriptive summary of daily ambient pollutants and meteorological factors in Nanjing, China, from 2013 to 2021.

Variables	*Mean*	*SD*	*Min*	*P_25_*	*M*	*P_75_*	*Max*	Excessive days	Excessive rate (%)
Ambient pollutants									
O_3–8h_ (μg/m^3^)	99.93	47.31	8.40	63.35	92.56	130.33	316.00	425	12.93
PM_10_ (μg/m^3^)	86.79	54.08	6.36	49.29	74.22	109.89	451.33	349	10.61%
PM_2.5_ (μg/m^3^)	48.93	35.37	4.64	25.29	38.89	62.21	327.44	565	17.19
SO_2_ (μg/m^3^)	16.44	13.51	3.07	7.86	12.10	20.60	139.44	0	0
NO_2_ (μg/m^3^)	42.86	19.36	5.64	28.50	38.89	53.75	140.67	171	5.20
CO (mg/m^3^)	0.91	0.33	0.29	0.70	0.85	1.04	2.99	0	0
Meteorological factors									
Mean temperature (°C)	16.94	9.04	−6.70	9.00	17.70	24.60	34.50	–	–
Mean relative humidity (%)	72.89	14.43	28.00	63.00	73.00	84.00	100.00	–	–

#### 3.1.2 The changing trend of cardiovascular disease deaths

Table 2 provides a summary of daily resident deaths of cardiovascular disease in Nanjing from 2013 to 2021. The number of deaths attributable to cardiovascular disease increased from 15,543 to 18,733. In total, there were 151,609 deaths, accounting for 43.87% of non-accidental deaths. Among these, 76,563 deaths (50.50%) were male and 75, 046 deaths (49.50%) were female. The number of male deaths was 76,563 (50.50%). Additionally, of the total deaths, 11.86% (17,976) of the deaths were aged 65 years or younger, while 88.14% (133,633) of the deaths were over 65 years old. On average, there were 46.12 daily deaths from cardiovascular disease (as shown in Figure 1).

**Table 2:** Summary of daily deaths related to cardiovascular disease among residents in Nanjing from 2013 to 2021.

Health indicators (cases/d)	*Mean*	*SD*	*Min*	*P_25_*	*M*	*P_75_*	*Max*
Cardiovascular disease	46.12	10.89	20	38	45	53	98
Male	23.29	6.39	7	19	23	27	54
Female	22.83	6.42	7	18	22	27	55
≤65 y	5.47	2.36	0	4	5	7	16
>65 y	40.66	10.25	15	33	40	47	88
Warm	41.02	8.75	20	35	40	46	98
Cold	51.25	10.41	24	44	51	58	89

### 3.2 Correlation analysis

**Figure 2:**
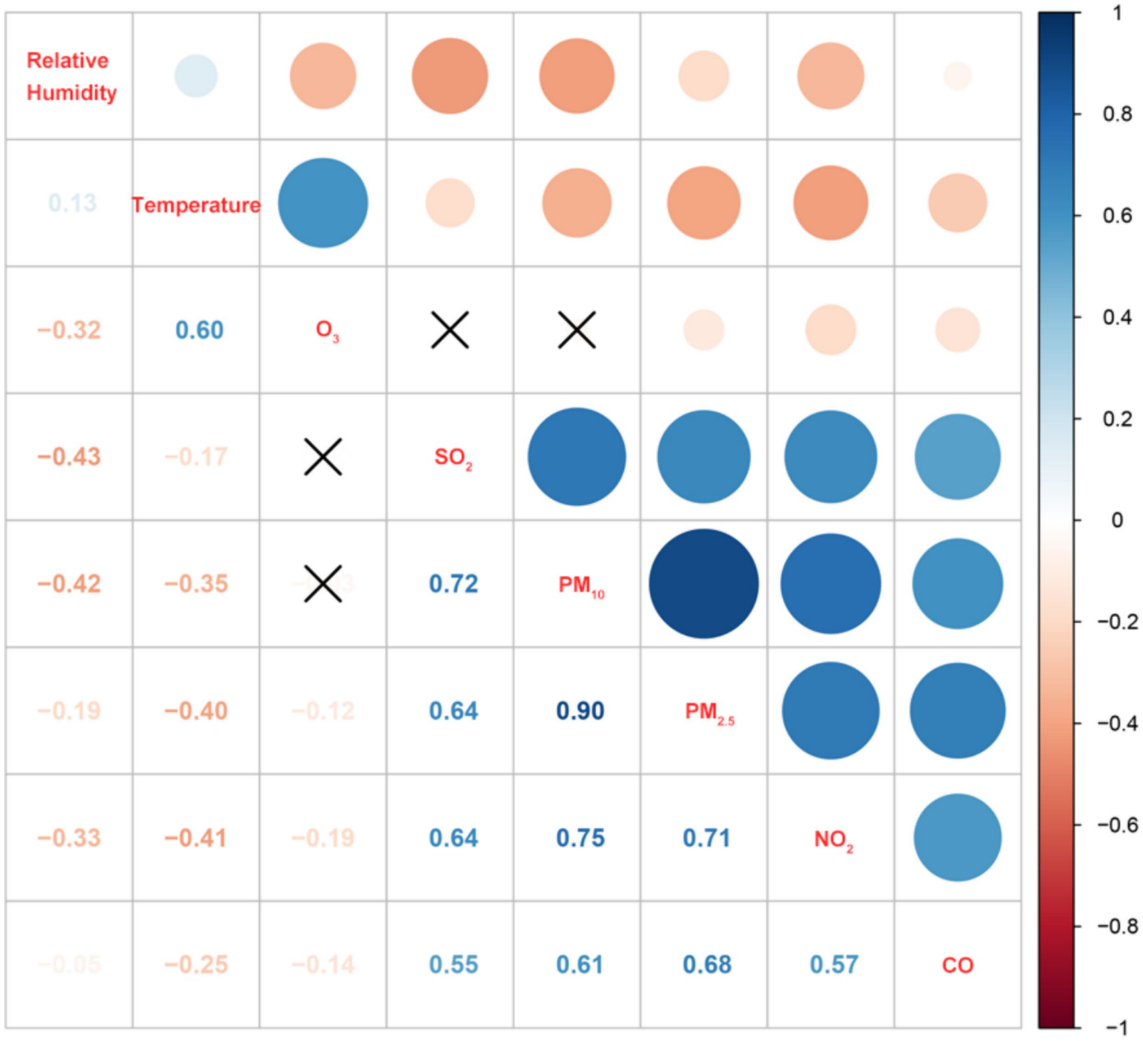
Spearman’s correlation between ambient pollutants and meteorological factors. ×, represents *p* > 0.05.

The results of the correlation analysis revealed that O_3_ exhibits weak negative correlations with PM_2.5_, NO_2_, CO, and relative humidity, but has a moderate positive correlation with temperature (0.60). Meanwhile, PM_2.5_, PM_10_, SO_2_, NO_2_, and CO were strongly correlated with each other. Furthermore, both temperature and humidity presented an inverse correlation with all the variables (Figure 2 and Supplementary Table S1).

### 3.3 Exposure-response assessment

**Figure 3:**
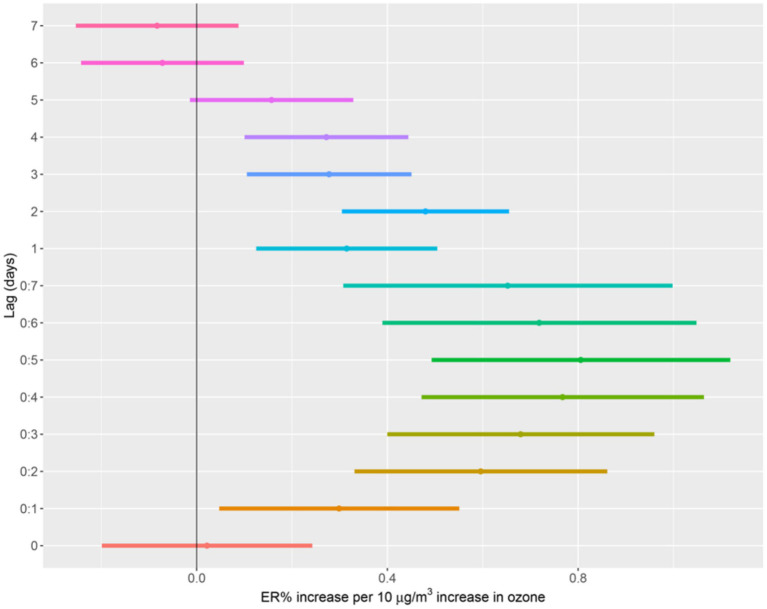
The ER% of CVD deaths associated with a 10 μg/m^3^ increase in O_3_ with the multi-pollutant model. Adjust for temperature, humidity, Holiday and DOW.

In the single-pollutant model, an increase of 10 μg/m^3^ in O_3_ was significantly associated with 0.77% (95%CI: 0.46, 1.08%) increase in cardiovascular disease deaths. After correcting for the other five gaseous pollutants (PM_2.5_, PM_10_, SO_2_, NO_2_, CO) in the two-pollutant models, the correlations between O_3_ and cardiovascular disease deaths remained significant and the ER% changed slightly. The correlations were enhanced marginally in the multi-pollutant models (Table 3). In the multi-pollutant model, the cardiovascular disease mortality risk increased by 0.48% (95%CI: 0.30–0.66%) for increments of 10 μg/m^3^ in O_3_ at lag 2; The effects of moving average lags were highest at lag05, with an ER% of 0.81% (95%CI: 0.49, 1.12%) (Supplementary Table S2). In addition, the data from 2013-2018 were analyzed to exclude the impact of the COVID-19 pandemic on cardiovascular deaths, and the similar results were obtained (Supplementary Tables S3, S4). Figure 3 illustrates the excessive risk (ER) and 95% confidence interval (95%CI) of cardiovascular disease from 0 to 7 days in a multi-pollutant model.

**Table 3:** The excess risk (95%CI) of CVD deaths associated with 10 μg/m^3^ increase of O_3_ with different models.

Pollutant	Models	ER% (95%CI)
	Single-pollutant model	
O_3_		0.77 (0.46, 1.08)
	Two-pollutant models	
O_3_	+PM_10_	0.75 (0.44, 1.06)
	+PM_2.5_	0.72 (0.41, 1.03)
	+SO_2_	0.78 (0.48, 1.09)
	+NO_2_	0.79 (0.48, 1.10)
	+CO	0.77 (0.48, 1.09)
	Multi-pollutant model	
O_3_	+PM_10_+ PM_2.5_+ SO_2_+ NO_2_+ CO	0.81 (0.49, 1.12)

Adjust for temperature, humidity, Holiday and DOW.

**Figure 4:**
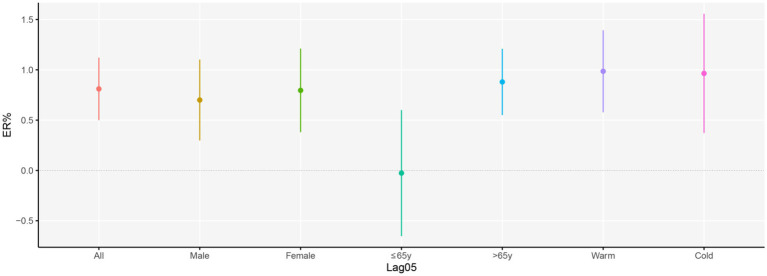
Excess risk (95%CI) in CVD deaths per 10 μg/m^3^ increase in O_3_ at lag 05 days, stratified by age group, sex, and seasons. Adjust for temperature, humidity, Holiday and DOW.

The stratified analysis revealed that the cardiovascular disease mortality risk of females was higher than males, although these differences were not statistically significant. In general, we observed that females (ER: 0.80, 95%CI: 0.38, 1.21%) had a higher vulnerability to ozone pollution compared to males (ER: 0.70, 95%CI: 0.30, 1.10%). In addition, the older group (ER: 0.88, 95%CI: 0.55, 1.21%) was found to have a significantly higher cardiovascular disease mortality risk, while the younger age group (ER: −0.03, 95%CI: −0.65, 0.60%) did not show any significant association. An analysis of season-specific effects revealed varying results. In warm seasons, the cardiovascular disease mortality risk (ER: 0.99, 95%CI: 0.58, 1.39%) was slightly higher than in cold seasons (ER: 0.96, 95%CI: 0.37, 1.56%) with no significant differences (Figure 4 and Supplementary Table S5).

### 3.4 Excess death attributed to ozone exposure

**Figure 5:**
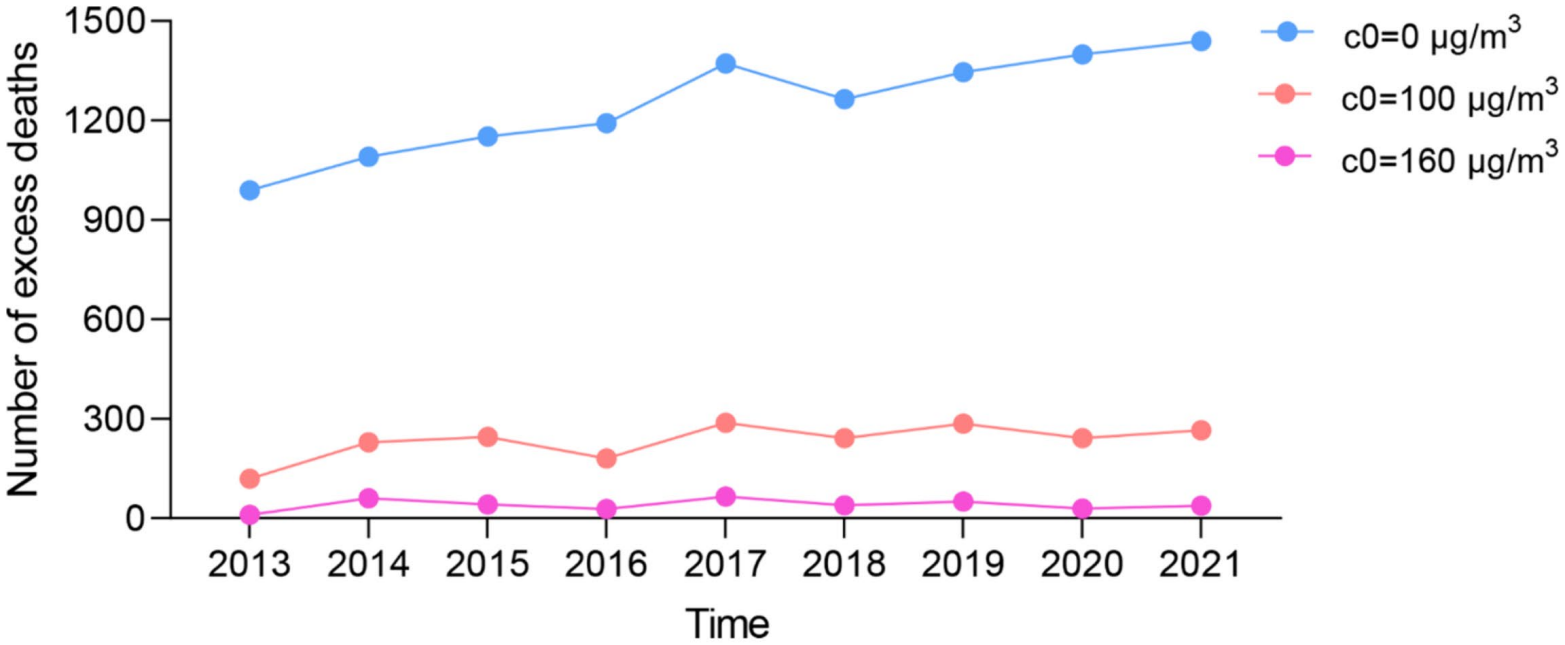
Excess deaths caused by ambient ozone pollution in Nanjing from 2013 to 2021.

Figure 5 displays the number of cardiovascular disease deaths attributed to ozone exposure in Nanjing, China. Between 2013 and 2021, the adoption of the domestic standard (160 μg/m^3^) for ozone reference concentration, ozone pollution contributed to 360 excess deaths from cardiovascular disease. There were 2,096 excess deaths due to ozone exposure when using the AQG guidelines (100 μg/m^3^), with a 123.20% increase in the number of excess deaths observed in 2021 compared to 2013. In a hypothetical scenario with a minimum level of ozone concentration set at 0 μg/m^3^, the attributable deaths would escalate to 11,242 cardiovascular disease deaths caused by ozone exposure.

If the ozone concentration were to be reduced to WHO standards, there would be 1,736 fewer deaths attributable to this pollutant, while there would be 10,882 fewer deaths if the minimum threshold was reached. For further details, please refer to Figure 5 and Supplementary Table S6.

### 3.5 Sensitivity analysis results

In sensitivity analyses, adjusting for the degree of freedom and lag days had minimal impact on the estimates for the associations between ozone exposure and cardiovascular disease deaths. When the degree of freedom was adjusted to 6, the excessive risk percentage (ER%) decreased, while it increased when the degree of freedom was adjusted to 8. However, when lag days were adjusted to 14, the ER% remained unchanged (Table 4). These stable results indicate that the model is consistent and unaffected by adjustments in the degree of freedom and lag days.

**Table 4:** The ER% between ozone and CVD deaths with different parameters in multi-pollutant model.

lag	df	Cardiovascular disease
7	6	0.68% (0.38, 0.98%)
	7	0.81% (0.49, 1.12%)
	8	0.85% (0.53, 1.16%)
14	7	0.81% (0.49, 1.12%)

Adjust for temperature, humidity, Holiday and DOW.

## 4 Discussion

This study aimed to examine the association between ozone exposure and excess deaths from cardiovascular diseases in Nanjing, China, from 2013 to 2021. Our findings provide further evidence supporting the positive association between ozone exposure and cardiovascular disease deaths by using a multi-pollutant model. Furthermore, we estimated the excess deaths of cardiovascular diseases caused by ozone exposure in Nanjing by analyzing local data and obtaining exposure-response relationship coefficients.

According to the Bulletin of the State of China’s Ecological Environment, there was an upward trend in ozone concentration in cities across the country from 2013 to 2019. However, the ozone concentration in 2020 was slightly lower than that in 2019.^3^ In Nanjing, the annual average concentration of ozone followed a similar pattern, increasing from 2013 to 2019 and then declining from 2020 to 2021. This trend aligns with the national pattern of ozone concentration. With rapid urbanization, energy consumption, and urban construction are usually accompanied by ambient pollution. The growing number of motor vehicles has resulted in increased nitrogen oxides emission, contributing to an increase in ozone concentration. Nanjing City, an economically developed city located in southeastern China, has responded to this issue by promoting various measures to reduce ozone concentration. For instance, improving the industrial structure; encouraging the use of green and clean energy; and expanding the utilization scale of solar energy, wind energy, biomass energy, and renewable energy sources. The implementation of new energy vehicles has made a significant contribution to curbing ozone concentrations by decreasing nitrogen oxide emissions.

In our study, we observed a lag effect in the association between ozone exposure and cardiovascular disease deaths. The magnitude of the association was lower than previous findings (0.98, 95%CI: 0.59, 1.38%) in 13 cities of Jiangsu Province in China (15). Liang et al. also found that each 10 ppb increment in MDA8 O_3_ concentration was associated with an increase in the risk of incident total CVD (1.07 [1.02–1.13]) (16). Meanwhile, a 10 μg/m^3^ increase in ozone was linked with a 0.59% (95%CI: 0.30–0.88%) elevated risk of death from cardiovascular diseases in Guangzhou, a southern city in China (17), and a 0.76% (95%CI: 0.21–1.32%) increase in Shenyang, a northern city in China (18). The effect of ozone exposure on cardiovascular diseases mortality in Nanjing remained constant, similar to the north and east cities of China, while it was higher than in the south. Populations in Northern China were found to have a significantly higher risk of mortality due to cardiovascular disease related to ozone exposure, as reported in our study (19). Furthermore, several meta-analyses have reported a positive association between short-term exposure to ozone and cardiovascular diseases (20, 21).

These studies primarily focused on analyzing the association between ozone and cardiovascular diseases using single-pollutant or two-pollutant models. However, considering that humans are typically exposed to multiple pollutants simultaneously, it is crucial to correct the influence of other pollutants. In our study, we observed that the association between ozone and cardiovascular diseases could either weaken or strengthen when analyzing two-pollutant models. This suggests that the presence of other pollutants can have a synergistic effect that influences the role of ozone. A case-crossover study analyzing air pollution and acute otitis media in children found that OR became slightly increased after adjusting for ozone (22). Similarly, Liu et al. conducted a national cohort study on air pollution and lung cancer, demonstrating that effect estimates for warm-season ozone were stronger in a multi-pollutant model (23).

The mortality and morbidity rates of cardiovascular diseases are rising, with ischemic heart disease and stroke ranking as the top-ranked causes of DALYs (Disability-Adjusted Life Years) in the 50 years and older group (24). In Nanjing, from 2013 to 2019, 88.14% of reported cardiovascular diseases occurred in the older individuals. Ozone, with its high oxidizing capacity, may induce oxidative stress damage and an inflammatory response upon inhalation (25). A study reported that ozone exposure was associated with adverse changes in blood pressure, cholesterol levels, glucose concentration, and body mass index (26). Wang et al. discovered that acute ozone exposure had a significant decreasing effect on high-frequency band of heart rate variability, which could potentially lead to acute cardiac events (27). Another study by Lei Tian et al. concluded that ozone exposure promoted cardiomyocyte apoptosis in a mitochondrial-dependent manner (28). This biological process may elucidate the unfavorable health outcomes of ozone on mortality related to the circulatory system.

In the age-stratified analysis, we found that the association between ozone exposure and cardiovascular diseases deaths was more pronounced in individuals aged 65 years and older compared to those under 65 years. Our results are supported by several studies (29, 30). It is widely accepted that the older people are at a higher risk and more susceptible to cardiovascular diseases. Cardiovascular diseases are a prevalent chronic condition caused by the accumulation of adverse factors such as hypertension, hyperlipidemia, and obesity (31, 32). Moreover, the decreased immune system function in older individuals makes them a vulnerable group. In our study, we observed that females had a higher risk of cardiovascular diseases mortality than males, which is consistent with previous research (33). The gender-specific vulnerability to ozone exposure may be influenced by differences in exposure levels and physiological factors. After the onset of menopause, women tend to experience an increase in cholesterol levels, whereas men’s cholesterol levels remain steady (34).

Furthermore, our study revealed that exposure to ozone was associated with higher mortality rates of cardiovascular diseases in warmer seasons compared to colder seasons (35, 36), with no significant differences. In both Chinese and US communities, the associations were found to be stronger in the southern regions during cool seasons as opposed to weaker associations in the northern regions during warm seasons. The European Approach Project reported a 0.46% (95%CI: 0.22, 0.73%) increase in the warm season, whereas insignificant estimates were observed during the cold season (37), the same as in Canada (33). Possible interpretations for the variation include the concentration of elevated ozone levels and the synergistic role between high temperature and ozone levels on death risk during the warm seasons (38). Additionally, the extremely cold winters in the northern regions limit the time people spend outdoors, potentially reducing the risk.

Understanding the increasing concentration of ozone is crucial in addressing excess deaths. In Nanjing, cardiovascular disease deaths attributed to ozone exposure have continued to rise from 2013 to 2021.

GBD report indicated an increase in disease burden due to ozone between 1990 and 2019 (24). In China, there were 373,500 (95% UI: 240,600–510,900) premature deaths attributable to cardiopulmonary causes related to ozone exposure (39). A projection study has estimated the fraction of deaths attributable to increased ozone concentration and indicated that higher ozone concentrations result in more deaths (40). This study highlights a discrepancy in health benefits when comparing the calculation using the WHO guideline and China’s standard. The ozone standards set by WHO (100 μg/m^3^), Europe (120 μg/m^3^), and the United States (National Ambient Air Quality Standards: 140 μg/m^3^) are more stringent than China’s standard (160 μg/m^3^) (41). It is evident that a reduction in ozone concentration to the levels recommended by WHO or even lower could potentially prevent more deaths. Therefore, China’s air quality standards should be more stringent to achieve greater health benefits.

## 5 Strengths and limitations

This study utilized a multi-pollutant model to assess the comprehensive health effects of combined exposure to multiple air pollutants and to account for the eliminated effect of high correlations among different pollutants. Additionally, our study evaluated excess deaths caused by short-term ozone exposure using a local exposure-response relationship coefficient, providing insights into the disease burden of local ozone pollution in Nanjing.

Nonetheless, this study is subject to certain limitations. Time series analysis, as an ecological research method, has inherent limitations. The exposure-response relationship derived from this analysis may be considered crude and may not capture all potential complexities. Furthermore, factors such as individual lifestyle choices and susceptibilities were not taken into account, which could influence the observed health outcomes.

## 6 Conclusion

Our study identified a significant association between ozone and cardiovascular disease deaths. Notably, older individuals and females exhibited a greater vulnerability to ambient ozone exposure. The number of deaths resulting from cardiovascular diseases due to ozone exposure rises in conjunction with increasing ozone concentration. Lowering the levels of ozone concentration down to WHO standards, or lower, would yield greater cardiovascular health benefits.

## Data availability statement

The original contributions presented in the study are included in the article/Supplementary material, further inquiries can be directed to the corresponding author.

## Ethics statement

The studies involving humans were approved by Ethics Committee of Nanjing Center for Disease Control and Prevention. The studies were conducted in accordance with the local legislation and institutional requirements. Written informed consent for participation was not required from the participants or the participants’ legal guardians/next of kin in accordance with the national legislation and institutional requirements.

## Author contributions

FS: Data curation, Writing – original draft, Conceptualization. XG: Formal analysis, Visualization, Writing – original draft. LW: Software, Data curation, Writing – original draft. YZ: Software, Data curation, Writing – original draft. MG: Supervision, Writing – review & editing. LX: Supervision, Writing – review & editing.

## Footnotes

^1^https://www.cma.gov.cn/zfxxgk/gknr/qxbg/

^2^http://cdc.cma.gov.cn/home.do

^3^https://www.mee.gov.cn/hjzl/
