# Supplementary material for: Assessing the impact of short-term ozone exposure on excess deaths from cardiovascular disease: a multi-pollutant model in Nanjing, China’s Yangtze River Delta
Source: Front Public Health. 2024 Jun 13;12:1353384. doi: 10.3389/fpubh.2024.1353384 (PMC11208627; doi:10.3389/fpubh.2024.1353384)
Supplement: Supplementary file 1 [file Table_1.pdf]

Supplementary Material

1 Supplementary Data

Table S1.

Spearman's correlation between Ambient pollutants and meteorological factors in Nanjing, China, from 2013 to 2021.

	O_3	$PM_{2.5}$	PM_{10}	SO_2	NO_2	CO	temp
$PM_{2.5}$	-0.12**						
PM_{10}	-0.03	0.90**					
SO_2	<0.01	0.64**	0.72**				
NO_2	-0.19**	0.71**	0.75**	0.64**			
CO	-0.14**	0.68**	0.61**	0.55**	0.57**		
temperature	0.60**	-0.40**	-0.35**	-0.17**	-0.41**	-0.25**	
relative humidity	-0.32**	-0.19**	-0.42**	-0.43**	-0.33**	-0.05**	0.13**

**, represents $P<0.001$.

Table S2.

The ER% of cardiovascular disease (CVD) deaths associated with a 10 μg/m^3 increase in O_3 with different lag days in a multi-pollutant model during 2013-2021.

Lag days	b	Se	ER% (95%CI)
0	0.000216	0.001126	0.02 (-0.20, 0.24)
1	0.003141	0.000966	0.31 (0.12, 0.50)
2	0.004786	0.000890	0.48 (0.30, 0.66)
3	0.002774	0.000878	0.28 (0.11, 0.45)
4	0.002716	0.000874	0.27 (0.10, 0.44)
5	0.001569	0.000873	0.16 (-0.01, 0.33)
6	-0.000720	0.000871	-0.07 (-0.24, 0.10)
7	-0.000830	0.000871	-0.08 (-0.25, 0.09)
01	0.002983	0.001280	0.30 (0.05, 0.55)
02	0.005940	0.001344	0.60 (0.33, 0.86)
03	0.006772	0.001419	0.68 (0.40, 0.96)
04	0.007644	0.001499	0.77 (0.47, 1.06)
05	0.008023	0.001585	0.81 (0.49, 1.12)
06	0.007158	0.001668	0.72 (0.39, 1.05)
07	0.006501	0.001750	0.65 (0.31, 1.00)

Adjust for temperature, humidity, Holiday and DOW.

Table S3.

The ER% of CVD deaths associated with a 10 μg/m^3 increase in O_3 with different lag days in a multi-pollutant model during 2013-2018.

Lag days	b	Se	ER% (95%CI)
0	0.000427	0.001400	0.04 (-0.23,0.32)
1	0.002481	0.001211	0.25 (0.01,0.49)
2	0.005717	0.001114	0.57 (0.35,0.79)
3	0.003932	0.001103	0.39 (0.18,0.61)
4	0.004212	0.001102	0.42 (0.21,0.64)
5	0.002629	0.001101	0.26 (0.05,0.48)
6	0.000413	0.001099	0.04 (-0.17,0.26)
7	-0.000140	0.001097	-0.01 (-0.23,0.20)
01	0.002499	0.001601	0.25 (-0.06,0.57)
02	0.006272	0.001674	0.63 (0.30,0.96)
03	0.007940	0.001776	0.80 (0.45,1.15)
04	0.009738	0.001892	0.98 (0.60,1.35)
05	0.010853	0.002019	1.09 (0.69,1.49)
06	0.010573	0.002143	1.06 (0.64,1.49)
07	0.010208	0.002260	1.03 (0.58,1.47)

Adjust for temperature, humidity, Holiday and DOW

Table S4.

The excess risk (95%*CI*) of CVD deaths associated with 10μg/m^3 increase of O_3 concentration with different models at lag 05 days during 2013-2018.

Pollutant	Models	ER% (95%CI)
	Single-pollutant model	
O_3		1.04 (0.65, 1.44)
	Two-pollutant models	
	$+PM_{10}$	1.04 (0.64, 1.44)
	$+ PM_{2.5}$	1.02 (0.63,1.42)
O_3	$+ SO_2$	1.07 (0.67,1.46)
	$+ NO_2$	1.07 (0.68,1.47)
	+ CO	1.07 (0.67,1.46)
	Multi-pollutant model	
O_3	$+PM_{10}+ PM_{2.5}+ SO_2+ NO_2+$ CO	1.09 (0.69,1.49)

Adjust for temperature, humidity, Holiday and DOW.

Table S5.

Excess risk (95%CI) in CVD deaths per 10 μg/m3 increase in O3 at lag 05 days during 2013-2021, stratified by age group, sex, and seasons.

groups	Lag days	ER% (95%*CI*)
All	05	0.81(0.49, 1.12)
Male	05	0.70(0.30,1.10)
Female	05	0.80(0.38,1.21)
≤65y	05	-0.03(-0.65,0.60)
>65y	05	0.88(0.55,1.21)
Warm	05	0.99(0.58,1.39)
Cold	05	0.96(0.37,1.56)

Adjust for temperature, humidity, Holiday and DOW.

Table S6

Excess deaths caused by ambient ozone pollution in Nanjing from 2013 to 2021

year	Excess deaths by cardiovascular disease		
	Minimum level *	AQG guidelines*	China's standard*
2013	989	119	10
2014	1091	229	60
2015	1152	246	42
2016	1192	180	28
2017	1372	288	65
2018	1264	242	39
2019	1345	285	51
2020	1399	242	29
2021	1439	265	38
Sum	11242	2096	360

*, Minimum level refers to 0 μg/m^3, AQG guidelines refers to 100 μg/m^3, China's standard refers to 160 μg/m^3.
